# Generation and analysis of 9792 EST sequences from cold acclimated oat, *Avena sativa*

**DOI:** 10.1186/1471-2229-5-18

**Published:** 2005-09-01

**Authors:** Marcus Bräutigam, Angelica Lindlöf, Shakhira Zakhrabekova, Gokarna Gharti-Chhetri, Björn Olsson, Olof Olsson

**Affiliations:** 1 Department of Cell and Molecular Biology, Göteborg University, Box 462, 403 20 Göteborg, Sweden; 2 Department of Computer Science, Högskolan i Skövde, Box 408, 541 28 Skövde, Sweden

## Abstract

**Background:**

Oat is an important crop in North America and northern Europe. In Scandinavia, yields are limited by the fact that oat cannot be used as a winter crop. In order to develop such a crop, more knowledge about mechanisms of cold tolerance in oat is required.

**Results:**

From an oat cDNA library 9792 single-pass EST sequences were obtained. The library was prepared from pooled RNA samples isolated from leaves of four-week old *Avena sativa *(oat) plants incubated at +4°C for 4, 8, 16 and 32 hours. Exclusion of sequences shorter than 100 bp resulted in 8508 high-quality ESTs with a mean length of 710.7 bp. Clustering and assembly identified a set of 2800 different transcripts denoted the *Avena sativa *cold induced UniGene set (AsCIUniGene set). Taking advantage of various tools and databases, putative functions were assigned to 1620 (58%) of these genes. Of the remaining 1180 unclassified sequences, 427 appeared to be oat-specific since they lacked any significant sequence similarity (Blast *E *values > 10^-10^) to any sequence available in the public databases. Of the 2800 UniGene sequences, 398 displayed significant homology (BlastX *E *values ≤ 10^-10^) to genes previously reported to be involved in cold stress related processes. 107 novel oat transcription factors were also identified, out of which 51 were similar to genes previously shown to be cold induced. The CBF transcription factors have a major role in regulating cold acclimation. Four oat CBF sequences were found, belonging to the monocot cluster of DREB family ERF/AP2 domain proteins. Finally in the total EST sequence data (5.3 Mbp) approximately 400 potential SSRs were found, a frequency similar to what has previously been identified in *Arabidopsis *ESTs.

**Conclusion:**

The AsCIUniGene set will now be used to fabricate an oat biochip, to perform various expression studies with different oat cultivars incubated at varying temperatures, to generate molecular markers and provide tools for various genetic transformation experiments in oat. This will lead to a better understanding of the cellular biology of this important crop and will open up new ways to improve its agronomical properties.

## Background

*Avena sativa *(oat) belongs to the *Poaceae *family. Other cereals in this family are wheat, barley and rye [1]. Wild oats are diploid, but all cultivated oats are hexaploid with an estimated 1C genome size of 13.23 pg, corresponding to about 13000 Mbp [2]. The commercial value of oat is derived both from its high-energy grain and from superior break-crop benefits. Oat plantations also have a comparatively low input demand of insecticides, fungicides and fertiliser due to high disease tolerance and low nourishment requirements of this crop [3]. Today oat is mainly used as animal feed, but it is one of the most promising future cereals in the functional food area. It has unique and well-documented cholesterol lowering effects, as a result of its soluble dietary fibres and high β-glucan content [4-6]. An oat diet greatly improves the well feeling of persons with celiac disease and also reduces the risk of diet-related diseases [7-9]. Oat is rich in natural phenolic antioxidants [10-14], which prevent the development of cardiovascular disease and certain cancers. In Sweden most of the harvested oat is used for animal feed. Only about 35000 tons per year are used for human consumption. However, due to its many health-enhancing properties, the market for oat and processed oat products like oat milk for human consumption is rapidly growing.

Many European countries grow winter oat, i.e. oat that is sown in the autumn and survives the winter in the field. Winter oat therefore has a longer growth season compared to summer varieties, allowing an earlier harvest and giving a higher yield. However, inherently oat is not as winter hardy as rye, wheat and barley. Due to the harsh climate in the Scandinavian countries, winter oat is therefore not grown there. Based on the English experience with winter oat, a Swedish winter oat would probably increase the yield of the harvest by at least 30% (John Valentine, IGER, UK, personal communication). In addition, since oat is the most important rotation crop for wheat and oil crops, an early harvest of winter oat would mean an earlier sowing also of the rotation crops, resulting in increased yields also for these crops. To develop a winter oat suitable for the Scandinavian climate is therefore of high priority (Anders Jonsson, The SwedishFarmers Supply and Crop Marketing Co-operative, personal communication). Since cold hardiness is a quantitative trait controlled by several genes [15], the traditional plant breeding programs have so far been of limited success in improving the cold hardiness for any of the important crop species [16] and the Swedish oat breeders have more or less given up their efforts to produce a Swedish winter oat (Rickard Jonsson, Svalöf Weibull AB, personal communication).

A cost efficient and rapid way to obtain new data from an organism with a large, complex and unknown genome is through partial sequencing of randomly selected cDNA clones. The resulting collection of expressed sequence tags (ESTs) reflects the level and complexity of gene expression in the sampled tissue and will also give an insight into gene structure of the chosen organism. This not only leads to the identification of a number of genes from the new organism that have known or putative functions but also to the discovery of completely novel, previously unknown putative proteins.

In this work, starting from 9792 EST sequences, we identified 2800 putative oat genes, several of which showed similarities to genes previously defined as cold stress related or involved in transcriptional regulation, signal transduction or metabolism. Several sequences that could represent new, unknown and unique oat genes were also identified. This data will now be used to study cold-acclimation in oat, to identify key genes in regulating winter survival, to produce molecular markers to facilitate the breeding for winter hardiness and to construct transgenic oat with increased freezing tolerance. These experiments will increase our general knowledge about the physiology of cold acclimation in plants in general and in oat in particular and in the long term allow the development of a Scandinavian winter oat.

## Results

### Cold-acclimation

**Figure 1:**
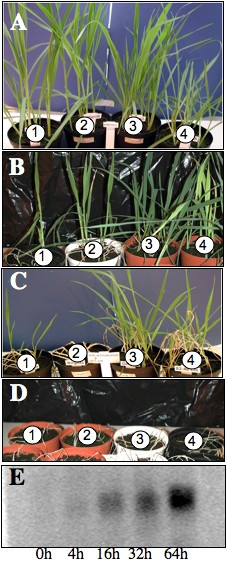
**Cold acclimation in oat**. (A) Four-week old Matilda (1) and Birgitta (2) (spring oat), Gerald (3) and 83-48-CH (4) (winter oat) grown in green house. (B) Plants recovered for one week in green house after acclimation (24 h at +4°C) and incubation at -15°C for 3 h. (C) Plants recovered for one week in the green house after acclimation (24 h at +4°C) and incubation -15°C for 6 h. (D) Plants recovered for one week in green house after acclimation (24 h at +4°C) and incubation at -15°C for 12 h. (E) In a separate experiment four-week old Gerald plants were incubated at +4°C for 0, 4 h, 16 h, and 64 h (indicated below lanes), total RNA was isolated and Northern hybridisations was performed using the oat Cor410 genes as a probe.

120 individual, four-week old, greenhouse-grown plants of the winter oat varieties Gerald, 83-48 CH and the spring oat varieties Matilda and Birgitta were incubated in the dark at +4°C (± 0.5°C) for 12 and 24 h and thereafter moved to -15°C for 1, 2, 4, 8 or 16 h. Another 120 individual plants were transferred directly at -15°C for 1, 2 4, 8 or 16 h. After the incubation period the plants were transferred back to the greenhouse and allowed to recover for one week. Visible freezing damage was then scored on a scale from 1 to 5, where 1 means no visible damage and 5 means a dead plant (Figure 1A–D). The damage was less severe on the plants that were incubated at +4°C prior to freezing than on plants directly transferred to -15°C (Table 1). Thus, oat plants, as expected, are able to cold-acclimatise to some extent. A clear difference could be seen between summer and winter varieties, however, the latter being more cold adaptable and more winter hardy both before and after acclimation (Table 1).

To confirm cold acclimation on the molecular level a known marker for cold acclimation was investigated. Total RNA was isolated from 3 week-old plants of the winter variety Gerald after incubation at +4°C for 4, 16, 32 and 64 hours. An oat sequence, corresponding to the wheat COR410 gene, known to be cold inducible in wheat [17], was amplified by PCR from genomic oat DNA. A northern analysis using RNA isolated from cold induced plants and oat COR410 as a probe, showed that the COR410 gene expression could be detected after 4 h and then remained strongly expressed even after 64 h of cold incubation (Figure 1E).

### EST sequencing and UniGene set construction

**Figure 2:**
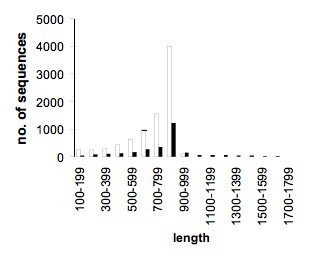
**Sequence length distributions**. Open bars, all 8508 sequenced ESTs; filled bars, the 2800 UniGene sequences. Average lengths were 710,7 and 800,1 bp, respectively. The longest sequences were 1017 and 2639 bp, respectively.

Since we aimed for both early and late cold inducible genes in our EST analysis, a cDNA library was constructed from pooled RNA samples isolated from oat plants at different stages of cold acclimation. After plating the library, bacterial colonies were randomly picked and 9792 single-pass sequence reactions performed on cDNAs present in plasmids from these clones. This resulted in 8508 high-quality ESTs of 100 bp or longer, with an average sequence length of 710 bp. The 8508 ESTs were assembled into 1100 contigs and 2616 singletons, giving 3716 candidate genes. All ESTs that contained rRNA, mitochondrial or chloroplast sequences were excluded from further analysis. Finally, the number of redundant sequences in the candidate gene set was eliminated. This was done by a comparative BlastX analysis of the candidate gene set to the NCBI non-redundant (nr) protein database (see "Materials and Methods"). This reduced the candidate gene set to 2800 transcripts with an average sequence length of 800 bp (Figure 2). These final genes were denoted as the AsCIUniGene (*Avena sativa *cold induced UniGene) set.

### Annotation and functional classification

The annotation of the AsCIUniGene set is based on homology. Each gene in the AsCIUniGene set inherited the annotation from the best match found after a BlastX search against nr protein database at NCBI. An expectation value (*E *value) threshold of 10^-10 ^was used. All sequences, in total 427, that had *E *values above this threshold were annotated as unknown.

**Figure 3:**
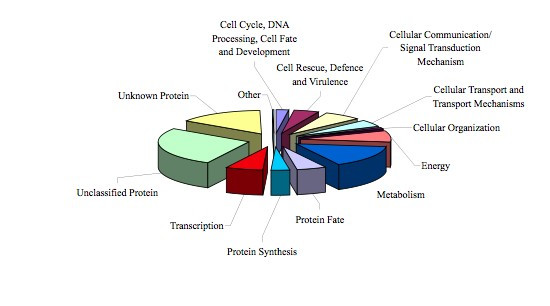
**Functional classification of the AsCIUniGene Set**. Classification was done both manually and according to the MATDB classification scheme (see Methods). The functional category is indicated by the text associated to respective piece in the diagram. The size of each piece is proportional to the relative abundance to the proteins assigned to this group.

The AsCIUniGene set was functionally classified by identifying every individual sequence in the set by the protein in the Munich Information Centre for Protein Sequences (MIPS) *Arabidopsis *database (MATDB) that gave the highest BlastX score. Since every gene in MATDB is assigned to at least one of the 28 different classes defined by the MIPS functional classification system, this proved to be a fruitful way to assign putative functions for sequences in the AsCIUniGene set. One drawback with the MIPS classification system is that it is somewhat crude since many of the functional classes were assigned automatically. Therefore, in addition to the automated process, we manually inspected the functional classification assignments for all the proteins and re-assigned them if necessary. In this way all of the 2373 oat sequences, out of which approximately 400 were manually assigned, were finally classified into the previously mentioned MIPS classes. The remaining 427 sequences (with *E *values > 10^-10 ^in the BLASTX search versus nr) could not be assigned either automatically or manually and where therefore classified as unknown proteins (Figure 3).

### The most abundant ESTs

To analyze the most abundant ESTs from the cold induced cDNA library we grouped the sequences by means of the KOG (Clusters of Eukaryotic Orthologous Groups of Proteins) database. This database currently includes 7 eukaryotic genomes, including *Arabidopsis*. This gave a functional annotation of sequences based on orthologous proteins [18]. In addition we used complementary databases to annotate our sequences like FOGs (Fuzzy Orthologous Group), which contains proteins with promiscuous domains that has not been assigned a KOG identity due to unclear orthologous relationships, TWOGs, which contains provisional clusters of proteins that are represented in two genomes and LSEs, which contains proteins that are lineage-specific expansions of paralogs present in the KOG database. The classification was based on best homology match of BlastX searches against *Arabidopsis *protein sequences where an expectation value (*E *value) threshold of 10^-10 ^was used. Proteins annotated in this way were termed "KOG-TWOG-LSE". Since not all *Arabidopsis *proteins are represented in the KOG database, not all ESTs could be annotated with a KOG-TWOG-LSE. Oat ESTs that had a homology match in *Arabidopsis *but not a KOG-TWOG-LSE annotation, inherited the annotation from MIPS annotation in MATDB. In addition, several of the sequences did not have an *Arabidopsis *homolog match with an *E *value above the threshold. These sequences were annotated with the best homolog match from a BlastX search against the nr protein database at NCBI. Again, an *E *value threshold of 10^-10 ^was used.

As a comparison, 2189 EST sequences from a non-induced oat leaf library [19] were analysed in the same way. As can be deduced from Table 2, the non-induced and the induced leaf libraries are quite different. In both libraries, chlorophyll a/b binding, ribulose 1, 5-bisphosphate carboxylase/oxygenase small chain and ribulose bisphosphate carboxylase/oxygenase activase (InterPro accession numbers IPR001344 and IPR000894) were the most expressed gene families, although in a different order (Table 2). The ribulose 1, 5-bisphosphate carboxylase/oxygenase sequence was the most common in the non-induced library, perhaps reflecting the higher photosynthetic activity in these plants. Interestingly, several cold related proteins were among the most abundant ESTs in the cold-acclimated library but were not represented among the most abundant ESTs from oat grown under optimal conditions. The cold-induced COR410 (Wcor410) is a dehydrin [17]. Dehydrins are expressed during water-deficit and cold stress. The cold-responsive LEA/RAB-related COR protein (Wrab17) belongs to group-3 of LEA-proteins and has previously been established as induced by cold [20]. Other interesting proteins in this context are the auqaporin PIP and hydroxyproline-rich glycoproteins. The aquaporins are membrane proteins that facilitate water transport across biological membranes. In *Arabidopsis *there are 13 members of aquaporins that belong to the plasma membrane intrinsic protein (PIP) subgroup. Recently, a study has shown that the PIP proteins are either up- or down-regulated in response to various abiotic stresses [21]. Hydroxyproline-rich glycoproteins have a strong homology to OSR40 proteins in *Oryza sativa*, which have previously been shown to be up-regulated by salt stress [22]. None of these genes were present in the non-induced library, indicating the great enrichment for cold stress related genes in the induced library. Since only genes that were annotated as cold induced, cold acclimated or as cold regulated were included, other stress related genes that indirectly also are involved in cold stress responses are most likely also enriched in this collection.

### Cold-regulated genes

In the functional classification sequences belonging to four categories; "Cell Rescue, Defence and Virulence", "Cellular Communication/Signal Transduction Mechanism", "Metabolism" and "Transcription" were considered to be of great potential interest for cold acclimation. Together these categories were represented by 931 entries in the AsCIUniGene set, corresponding to more than 30% of all genes (Table 3). To increase the resolution of this analysis and to improve the identification of putative cold-regulated genes we built a separate database with proteins previously reported in the literature to be directly involved in cold stress-related processes [23-26]. This "cold stress database" (CSDB) presently comprises 545 entries. In a BlastX search with the AsCIUniGene set against CSDB we identified 398 sequences in the AsCIUniGene set that showed significant homology (*E *≤ 10^-10^) to genes in the CSDB (Table 3). Thus, 14.2% of all the genes in the AsCIUniGene set seem to be cold stress related. Looking just at the two classes that we consider as most important for the cold acclimation process (Cell rescue, defence and virulence; Cellular communication and signal transduction) almost 40% of all sequences were homologous to potential cold stress related proteins. In the "Metabolism" and "Cellular Transport and Transport Mechanisms" classes, cold stress related genes were also overrepresented (approx. 25%).

To analyze whether this high proportion of cold regulated genes in relation to the total number of genes in the AsCIUniGene set was similar to other EST collections derived from cold acclimated plants, we downloaded and analysed EST datasets from cold acclimated wheat and barley. The datasets were clustered and assembled with the TGI clustering tool (see Material and Methods). This resulted in a TaCIUniGene set of 2894 genes and a HvCIUniGene set of 3932 genes. In addition, as a comparison the same oat ESTs collection derived from leaves of plants grown under optimal conditions (Table 2) was clustered and assembled into a AsNIUniGene set of 1445 sequences. These three UniGene sets were then searched against the CSDB. This showed that the proportions of cold stress related genes were 9.6% in the TaCI candidate gene set and 11.1% in the HvCI, but only 5.1% in the AsNI UniGene set (Table 4). Thus the AsCI UniGene set is quite different from previous oat EST collections and also the EST collection that contains the highest proportion of cold induced genes of all cereals.

### Phylogenetic analysis of AP2 containing proteins

**Figure 4:**
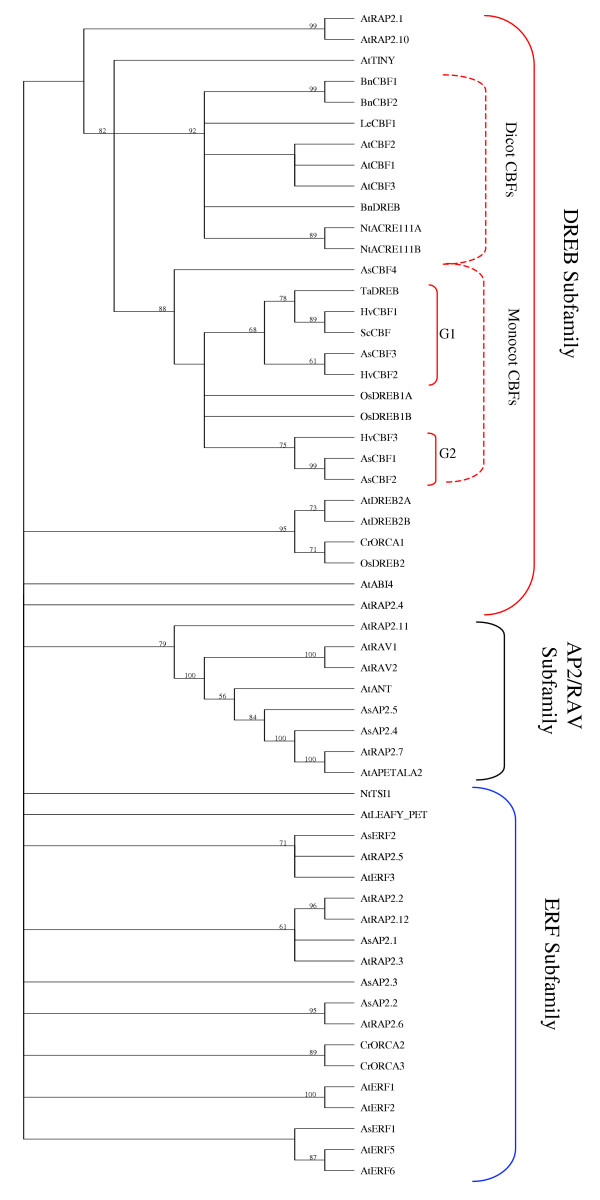
**Phylogenetic analysis of ERF/AP2 domains**. The tree was built by means of the Neighbour-Joining method using 56 different ERF/AP2 domains. Numbers along the branches correspond to bootstrap values after 1000 replicates. Branches without values have bootstrap values < 55. AC-numbers and source of the sequences are given in Table 6.

Among the 190 sequences in the transcription class (Table 3), 107 were found to be homologous to transcription factors. Remarkably, 51 of these 107 sequences were homologous to genes in the CSDB, representing almost 48% of all transcription factors found in the AsCIUniGene set. To investigate this a bit more, the transcription factors were further classified into 14 different transcription factor families (Table 5). Dominating among these were genes encoding AP2 domain, homeodomain and zink finger proteins. Proteins belonging to the CBF/DREB1 transcription factor family have previously been shown to be the regulators of the majority of cold-response genes. The CBF/DREB1 family belongs to the AP2/ERF super family [27] and in *Arabidopsis *the AP2/ERF super family comprises 145 proteins. Based on similarities in their DNA-binding domains, these proteins have been subdivided into the AP2, RAV, DREB and ERF subfamilies and one family with the remaining proteins. In the AsCIUniGene set, we found 11 sequences belonging to the AP2/ERF superfamily (Table 5). The AP2/ERF domain of these 11 *Avena *proteins were more closely analysed and also compared to 45 previously described AP2/ERF containing proteins [28]. The resulting phylogenetic tree revealed that 4 of the oat AP2/ERF proteins belonged to the DREB subfamily, 2 to the AP2/RAV subfamily and the remaining 5 sequences to the ERF subfamily (see Figure 4 and Table 6). From the analysis it can also be deduced that the oat CBF/DREB1 proteins are most closely related to the monocot CBF/DREB1 proteins (Figure 4).

**Figure 5:**
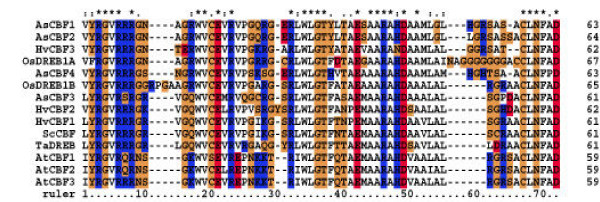
**ClustalW analysis of AP2 DNA-binding domains**. AP2domain of AsCBF1, AsCBF2, AsCBF3 and AsCBF4 were aligned to AP2 domains from CBF related proteins from other plants as indicated in the text to the left. *Arabidopsis thaliana *AtCBF1, AtCBF2, AtCBF3; *Horedeum vulgare *HvCBF1, HvCBF2, HvCBF3; *Triticum aestivum *TaDREB; *Oryza sativa *OsDREB1A, OsDREB1B; *Secale cereale *ScCBF (AC-numbers of the sequences are given in table 6). The amino acid residues are coloured based on the functionality, and the colouring schema is as follows: Acidic residues (DE) are red, basic residues (RHK) are blue, hydrophobic residues (AILMFPV) are white and hydrophilic residues (NCQGSTY) are orange.

To further analyse relations between oat and other monocot CBF/DREB1 proteins, a multiple alignment of AP2/ERF domains from AsCBF1, AsCBF2, AsCBF3, AsCBF4, OsDREB1A, OsDREB1B, HvCBF1, HvCBF2, HvCBF3, ScCBF and TaDREB was made. In addition, the Arabidopsis CBF/DREB sequences AtCBF1, AtCBF2 and AtCBF3 were included to further elucidate the relation between dicots and monocots in this respect (Figure 5). In previous studies it has been reported that in particular two amino acids, a valine at position 19 (V19) and a glutamic acid at position 24 (E24) (Figure 5) in the AP2/ERF domains of Arabidopsis have important roles in determining DNA-binding specificity [27]. The AsCBF proteins have the conserved valine in the V19 position but not the glutamic acid in the E24 position (Figure 5). Instead, a valine is conserved in this position. This feature is in fact shared among all included monocot CBF/DREB proteins (Figure 5). The monocot CBF/DREB1 proteins could be further divided into three subgroups (Figure 4). The first subgroup (G1) contained the AsCBF3, HvCBF1, HvCBF2, ScCBF, and TaDREB proteins, the second subgroup (G2) contained the AsCBF1, AsCBF2 and HvCBF3 proteins and the third subgroup the remaining AsCBF4, OsDREB1 and OSDREB2 proteins (Figure 4). This grouping is based on differences in aa at positions 10, 18 and 39 between the different proteins (Figure 4, Figure 5). At position 10 the G1 proteins have a basic arginine (R) residue, the G2 proteins a hydrophilic asparagine (N) and the G3 proteins N, hydrophilic serine (S) or glycine (G) residues (Figure 5). In position 18 there is a basic residue, an arginine (R) in G2 and G3 proteins whereas G1 proteins have a hydrophilic residue, a glutamine (Q) (Figure 5). Finally G2 proteins have a hydrophilic tyrosine (Y) in position 39 while in G1 and G3 proteins this position is occupied by a hydrophobic phenylalanine (F) except for AsCBF4, which has a basic histidine (H) (Figure 5).

### Expression of the *AsCBF *genes

**Figure 6:**
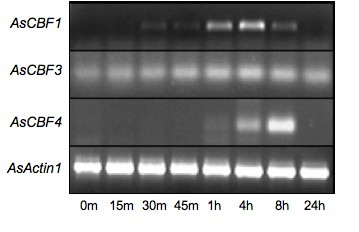
**RT-PCR analysis of *AsCBF *gene expression in three-week old oat**. Plants were incubated at +4°C for the times indicated below the lanes (m, minutes; h, hours). Total RNA was isolated from leaves and PCR reactions were run in 30 cycles using *AsCBF1*, *AsCBF3*, or *AsCBF4 *specific primers, as indicated to the left of the picture. Equal loading and RNA quality was controlled by amplification of the oat *AsActin1 *gene. A 1 kb ladder was used as a size marker (not shown).

To explore whether the four identified oat *AsCBF *genes were cold induced with similar kinetics as other previously described CBF genes an expression analysis was performed. Multiplex RT-PCR was run on total RNA isolated from leaves of three weeks old plants cold induced (to +4°C) at time points between 15 min to 24 h using gene specific primers for *AsCBF1*, *AsCBF2*, *AsCBF3 *and *AsCBF4 *respectively. Total RNA isolated from untreated plants at the same time points were used as a comparison. An oat actin gene (*AsActin*) was also amplified from the same RNA samples as a loading and RNA quality control. To define conditions where the PCR amplification was in the exponential phase, several experiments with 30, 35 or 40 cycles were performed. This showed that the different *AsCBF *genes all were induced by the cold treatment but that they were differentially regulated (Figure 6). The *AsCBF1 *gene was not detectable at time 0, was induced after about 30 min, peaked at 4 hours and was completely shut off after 24 h. The *AsCBF3 *gene had quite a different expression pattern. It was weakly expressed also in non-induced plants, but was in addition rapidly induced already at the first time point after 15 min. The expression levels continued thereafter to increase, peaked after about 4 hours but still showed an elevated expression at 24 h. The *AsCBF4 *gene was slowly induced and not detected at all until after 4 hours. Unlike the others its expression peaked a bit later, after 8 hours and then had completely declined after 24 h. Despite several attempts, using different primer pairs we could not obtain a reproducible expression pattern of the *AsCBF2 *gene (data not shown). Thus, the different oat *AsCBF *genes seem to be active during different phases of the cold acclimation process and therefore perhaps induce different downstream gene programs. We are presently addressing this issue more specifically.

### Identification of microsatellites

**Figure 7:**
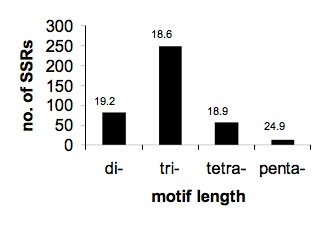
**Microsatellite distribution**. SSRs were sorted according to repeat motif length. The height of the bar indicates the number of SSRs that were found for each class. Numbers above bars denote average total SSR length.

Using the Sputnik program and threshold values as specified in the Methods section, we searched for potential microsatellite (SSR) sequences in the 3716-candidate gene set. In total, 399 di- to pentanucleotide SSRs that fulfilled the criteria of the search were identified. This corresponds to approximately one microsatellite per 13 kb of sequence. Using the same methods and thresholds, Cardle et al. (2000) found on average one SSR per 14 kb of EST data in *Arabidopsis*. This indicates that the SSR frequency in oat is similar to that in *Arabidopsis*. In the oat sequence collection, tri-nucleotide repeats were the most commonly found followed by di-nucleotide repeats (Figure 7), which again matches the results from *Arabidopsis *[29]. Except for two exceptional TA/AT SSRs of length 45 and 55 bp, SSR lengths ranged from 15 to 25 bp, with 16 and 17 bp being the most common.

Work is now in progress to determine which of these SSRs can be reproducibly amplified by PCR, are polymorphic, and can be linked to a phenotypic marker. The vast majority of the oat SSRs were found in non-coding DNA. Since they nevertheless represent actively transcribed genes, we expect that several of these will turn out to be useful markers for breeding.

## Discussion

Plant expressed sequence tags (ESTs) have proven to be valuable tools in molecular biology research and a number of collections from many different plant species are now publicly available [19]. In cereals, which are the most important food providers on earth, several major EST sequencing projects have been carried out. At the time of writing, there are 284 779 publicly available ESTs from *Oryza sativa *(rice), 562 786 from *Triticum aestivum *(wheat) and 367 768 from *Hordeum vulgare *(barley). In contrast, there are only 7 624 entries for *Avena sativa *(oat) and no sequences from cold acclimated oat are available. Obviously, there is a great need for more EST sequencing also on this important crop. Here we contribute an additional 9 792 sequences, originating from cold-acclimated oat, to the research community. Since we were mainly interested in genes involved in the perception, signal transduction and early regulation of cold acclimation, we focused on short incubation times from a few minutes to 24 h. Already after 12 hours acclimation, there was a clear difference in freezing tolerance between acclimated and non-acclimated plants and winter varieties were more tolerant than spring varieties (Table 1). To confirm that cold induced genes were overrepresented in these plants, a northern analysis was performed on an oat gene corresponding to the previously described cold induced wheat *COR410 *gene on RNA isolated from several different time points during cold acclimation at +4°C. This revealed that the diagnostic *COR410 *gene was cold induced also in oat and, interestingly, the peak expression level was higher in the winter varieties (Figure 1 and Table 1). The same tendency with earlier induction and higher expression levels was also seen for other cold induced genes (data not shown).

Pooled leaves from confirmed cold induced plants were used for cDNA construction and EST sequence generation. Since leaves were used as the RNA source, the most common ESTs in our collection represent various genes involved in photosynthesis, like chlorophyll a/b binding protein, ribulose 1, 5-bisphosphate carboxylase/oxygenase, ribulose bisphosphate carboxylase/oxygenase activase, photosystem I reaction centre protein, fructose-bisphosphate aldolase, carbonic anhydrase/carbonate dehydrase and photosystem II oxygen-evolving complex proteins. Other well-represented sequences are those encoding ribosomal proteins (Table 2). However, among the 20 most expressed gene families, dehydrin was also present, indicating that our collection indeed represents plants with a cold stressed induced condition. This was confirmed by a direct comparison to an EST set derived from leaves of non-induced plant. In this collection, dehydrin and other cold induced genes were not among the 20 most highly expressed.

From our cold induced EST collection, an AsCI UniGene set of 2 800 genes was identified. Of these, 1 726 could be placed into the functional groups defined by MIPS (Figure 3), leaving a relatively large proportion of the genes (approx. 40%) outside of this classification. Perception of the stress stimuli, transduction of the stress signal and a molecular response are necessary activities if the plant is to react to abiotic stress. In oat, however, very little is known about cold stress response at this level, although the general mechanisms are probably similar in all plants. In order to better identify oat genes involved in the cold response we therefore created a database denoted CSDB (cold stress data base), in which all genes available from the public domain and classified as cold stress responsive or cold induced were collected. When the sequences in the CSDB were compared to the oat AsCIUniGene set we found that 398 sequences matched, indicating that at least 14% of all the genes in the AsCIUniGene set are involved in cold stress. Among these, sequences encoding activities related to perception, signal transduction and transcriptional regulation were overrepresented (Table 3). From the oat EST collection generated from leaves of three weeks old oat plants grown under green house conditions we created a UniGene set of 1445 different transcripts using the same tools as with the AsCIUniGene set. This non-induced leaf set was denoted AsNIUniGene. When the CSDB was searched with AsNIUniGene only 5.1% of the genes were found to be similar (Table 4), a dramatic difference to the AsCIUniGene set. These studies were extended to EST collections from cold acclimated wheat and barley. By creating UniGene sets (TaCIUniGene and HvCIUniGene) both these collections were analysed in the CSDB. We then found that the amount of cold stress related genes were around 10% in both the wheat and barley collections (Table 4). Generalising, it seems like that at least 10% of all expressed genes in cold acclimating plants are devoted to various cellular responses needed to prepare the plant to freezing temperatures. The cold induced oat gene collection will now be a valuable new asset in further analysis of such genes.

Our functional analysis of the AsCIUniGene set showed that transcription factor genes were represented by 107 sequences, belonging to at least 14 different families (Table 5). Of these, 51 were homologous to cold-induced genes from other systems. Of special importance for cold acclimation is the CBF transcription factor family. Genes in this family regulate several different downstream genes, including the COR genes [16,30]. However, this regulation is complex and several different CBF genes are involved. From the AsCIUniGene set we identified four oat CBF genes, denoted *AsCBF1*, *AsCBF2*, *AsCBF3 *and *AsCBF4*. Our phylogenetic and multiple alignment analysis showed that all four belonged to the monocot DREB subfamily of ERF/AP2 domain proteins. The *AsCBF1 *and *AsCBF2 *genes were very closely related, while the *AsCBF3 *and *AsCBF4 *genes were somewhat more distantly related to each other and also belonged to a different clade than the *AsCBF1 *and *AsCBF2 *genes (Figure 4 and 5).

To investigate the expression profile of the *AsCBF *genes, we designed gene specific primers and by RT-PCR analysis showed that these genes indeed were cold induced, but that their expression patterns were different. Their expression ranged from early induction already after 15 min (*AsCBF3*) to induction after 1 h (*AsCBF4*) and from peaking at 4 h (*AsCBF1 *and *AsCBF3*) to peaking at 8 h (*AsCBF4*) (Figure 6). The *AsCBF3 *was particularly interesting since it was weakly constitutively expressed, showed a clear increase in expression after cold treatment and still expressed after 24 h. Despite several attempts using different primer pairs we could not obtain a reproducible expression pattern of the *AsCBF2 *gene. The reason for this is presently not known. The complex regulation of the *AsCBF *genes is different from what was previously described in *Arabidopsis *[31] where the *AtCBF1*, *AtCBF2*, and *AtCBF3 *genes follow more or less the same expression pattern with a rapid induction after 15 min and a peak after 2 h. This indicates that CBF factors have intricate and different individual roles in inducing and maintaining cold acclimation in oat. This is corroborated by preliminary data from barley. This cereal has at least 10 different genes encoding CBF factors, which are all differentially regulated (Eric Stockinger, Ohio State University, personal communication). Thus, a more detailed analysis of the structure and regulation of *CBF *genes in cereals may reveal new pathways of cold induction, not present in *Arabidopsis*.

A number of genes with hitherto unknown functions were identified in the AsCIUniGene set. These were divided into two groups, one in which homologous or similar genes from other systems exist and one where no significant similarities could be found to any other sequence, i.e. genes that could be oat specific. In order to rule out that small "non-real" peptides contributed to this group, only sequences with open reading frames of 100 aa or more were included. Work is now in progress to elucidate which of the 427 oat specific unknown genes that are induced by cold stress, drought stress or combinations of different stress factors. Assuming that approx. 10% of these sequences are cold related, more than 40 completely new oat genes involved in cold acclimation will be present in this collection. Such genes are potentially very interesting and could encode hitherto uncharacterised proteins or regulatory factors involved in cold-adaptation and freezing protection

Microsatellites (SSRs) are excellent DNA markers for genetic mapping, since they are polymorphic, abundant, show a co-dominant inheritance and are easy to analyse by PCR [32]. SSRs have therefore been widely utilized in plant genomic studies [33-36]. They are especially advantageous when there is a need to track desirable traits in large-scale breeding programs and when defining anchor points for map-based gene cloning strategies. However, only a few oat SSRs are currently available. Here we identify approximately 400 potential oat SSRs, the majority present in the non-coding part of the EST sequence. Work is now in progress to optimise primers for these SSRs and to identify the ones that give reliable PCR products and are polymorphic. Crude genetic maps have been developed for both diploid [37,38] and hexaploid oat [39], but these maps need to be improved [40]. The best SSR markers will therefore be mapped to the oat genome, and linked to valuable genetic markers.

The AsCIUniGene set will now be used to fabricate an oat biochip carrying all 2800 identified genes. In addition, by constructing subtractive libraries, more cold-related ESTs will be generated. Various expression studies will be performed and genes from our collection that show a rapid induction to either cold or drought will be selected for further analysis. We are especially interested in those genes in our EST collection that show a very rapid induction at +4°C and have DNA binding properties. Especially promising genes will be tested in transgenic *Arabidopsis *and oat systems [41,42] and by complementing chosen *Arabidopsis *T-DNA knock-out mutants.

## Conclusion

A UniGene set of 2800 genes was produced from a cold induced oat cDNA library. Further analysis revealed that genes related to cold stress were overrepresented in this library and that several genes could encode hitherto unknown functions. RT-PCR analyses of CBF transcription factor genes revealed that they are differentially expressed in oat and therefore might regulate different cold pathways. Approx. 400 potential SSR markers are also present in the collection, several in non-coding regions and in close vicinity to genes involved in regulating cold acclimation.

## Methods

### Plant growth

Oat plants, *Avena sativa *v. Gerald, 83-48 CH, SW Matilda and SW Birgitta were obtained from the SW-collection (Svalöf Weibull AB, Landskrona, Sweden). Gerald and 83-48 are English winter varieties while Matilda and Birgitta are Swedish spring varieties. Seeds were germinated in 2-liter pots filled with fertilized and pressed peat. Plants were cultured in a greenhouse under natural light supplemented with metal halogen lamps, giving a photon flux density of 240 μmol per m^2 ^per sec. The photoperiod was 18 h, the day/night temperature was 20/12°C and the relative humidity about 70%. The plants were watered as needed.

### Cold induction experiments

To investigate the cold acclimation capacity of the chosen oat varieties, 24 pots with 10 seeds each of Gerald, 83-48 CH, Matilda, and Birgitta were prepared. About three weeks after germination, when each plant had produced 3 – 4 leaves, pots were moved to a dark cold room at +4°C (± 0.5°C) and incubated for 12 and 24 hours. After this period the pots were moved to -15°C (± 1°C) for 3 h, 6 h and 12 h. In addition, plants were moved from the greenhouse directly to -15°C, and incubated for 3 h, 6 h and 12 h. After the cold incubation period, the plants were moved back to the greenhouse for recovery. One week later the cold damage was visually scored.

### Total RNA preparation

Winter oat (Gerald) was germinated and grown for three weeks in the greenhouse. They were then incubated in the dark at +4°C (± 0.5°C) for 4, 16, 32 or 48 hours. At every timepoint, leaves were randomly picked from several individual plants and pooled. RNA was extracted from pooled leaves essentially as described by Chang *et al. *(1993). Tissue isolates were ground in liquid nitrogen, transferred to 65°C CTAB extraction buffer (2% CTAB [hexadecyltrimethylammonium bromide] [Sigma], 2% PVP [polyvinyl pyrrolidone, intrinsic viscosity 29–32] [Sigma], 100 mM Tris-HCl [pH 8.0], 25 mM EDTA, 2.0 M NaCl, 0.5 g per L spermidine, 2% β-mercaptoethanol), and extracted twice in equal volumes of chisam (phenol:chloroform:iso-amyl-alcohol 1:1:24). RNA was precipitated overnight at 4°C by adding 0.25 v/v 10 M LiCl. The precipitate was dissolved in 1 × SSTE (1.0 M NaCl, 0.5% SDS, 10 mM Tris-HCl, 1 mM EDTA), pH 8.0, extracted with an equal volume of chisam, precipitated with two volumes 99.5% ethanol, and re-suspended in distilled water treated with DEPC. Total RNA of each sample was quantified spectrophotometrically at OD_260_. An OD_260 _of 1 corresponded to 40 μg/ml RNA. Subsequently, the RNA was precipitated and re-suspended in DEPC-treated distilled water to a final concentration of 1 mg/ml.

### Northern hybridization

Ten μg of total RNA were denatured with glyoxal/DMSO [43] and separated on a 1% agarose gel. The RNA was blotted onto a nylon membrane (Boeringer-Mannheim) and hybridized in Church hybridization buffer [44]. An oat sequence, similar to the wheat COR410 gene was used as a probe. This was isolated by PCR amplification from oat genomic DNA using the forward primer 5'-ATGGAGGATGAGAGGAGCAC-3' and the reverse primer 5'-TTTCTTCTCCTCCTCGGGC-3'. Primer design was based on the wheat sequence. Amplification resulted in an 530 bp sequence which was verified by DNA sequencing (data not shown). The fragment was labelled with ^32^P-dCTP (Amersham), using a random hexanucleotide mix and labelling-grade Kleenow enzyme (Boeringer-Mannheim). Stringency washes were performed at 65°C for 2 × 5 min in 2 × SSC, 0.5% SDS and for 4 × 5 min in 0.2 × SSC, 0.1% SDS. Membranes were exposed to X-ray film (Du Pont Medical Scandinavia AB).

### cDNA library construction and EST sequencing

Total RNAs isolated from plants incubated at +4°C for 6, 12 and 24 hours were pooled. The RNA pooled preparation was sent to MWG Biotech (Germany) where cDNA libraries were constructed, cDNA cloned into the pSPORT1 vector [45] and EST sequencing was performed.

### Bioinformatic tools

All similarity searches were batch executed locally using the BlastN, BlastX or TBlastX tools [46], all included in the BLASTALL program package [47]. Transeq, a program from the EMBOSS package [48,49] was used to translate DNA sequences into protein sequences. Conserved domain search (CD-search) was performed against the Conserved domain database (CDD) at NCBI [47] using the Reversed position specific Blast (RPS-BLAST) algorithm with translated ESTs. InterProScan [50,51] was used to scan translated ESTs for protein signatures in the InterPro member databases. For the multiple alignments we used ClustalW [52], included in the MacVector 7.2.2 package (Accelrys Inc). The phylogenetic tree was constructed by means of the MacVector 7.2.2 tool kit using the neighbour-joining (NJ) algorithm. Appropriate PERL scripts were written in order to pipeline the process of running tools in sequence, parsing result files and loading the results into the database. All data and results are stored in a PostgreSQL database.

### Data sets and treatment

In this paper started off with four different primary sequence data sets. The first set was the 9792 ESTs from cold acclimated oat, which was denoted the *Avena sativa *Cold Induced (AsCI) data set. The second data set comprises 2189 ESTs [53], which originated from untreated green leafs of 3 weeks old oat plants and was denoted the *Avena sativa *NonInduced (AsNI) data set. The third data set, which contains 4337 sequences originates from cold acclimated wheat [53] was denoted the *Triticum aestivum *Cold Induced (TaCI) data set and the final data set comprises 5418 sequences from cold stressed barley plants [53] and was denoted the *Hordeum vulgare *Cold Induced (HvCI) data set.

### EST clustering and assembly

The AsCI data set was filtered, clustered and assembled with the Paracel Transcript Assembler™ (PTA) program (Paracel, Pasadena, CA), which integrates quality filtering, clustering, and assembly into a single pipeline. The filtering step includes masking of vector sequence, low-complexity, low-quality, repeats and poly(A) regions. In the next step clustering was performed. Here PTA utilizes the Haste algorithm in an all-versus-all sequence comparison. The criterion set for clustering sequences together was an alignment of a minimum of 100 bases and with at least 93% similarity between the aligned sequences. Sequences that did not fit into such clusters were defined as singlets. In the assembly step PTA uses CAP4, which is a refinement of the CAP3 algorithm [54]. Sequences that did not fit into a contig were also defined as singlets. Finally, ESTs in singlets that had passed the filters but had an unmasked sequence < 100 bases were discarded. The resulting singlets and contigs represented the AsCI candidate gene set.

The other data sets where clustered and assembled into candidate gene sets using the TGI clustering tool [55]. The clustering was performed by a slightly modified version of NCBI's MegaBlast program [56] and the resulting clusters were assembled using CAP3.

### Most abundant ESTs

Individual ESTs were first annotated by the best BlastX homolog match against *Arabidopsis thaliana*, where an *E *value of < 10^-10 ^was used. The *A. thaliana *proteins were retrieved from the MIPS *Arabidopsis *database (MATDB). Thereafter the annotation given in the KOG database [18] was retrieved for each *A. thaliana *protein. For those *A. thaliana *homologs that did not have a KOG annotation, the EST sequence inherited the annotation from MATDB. Those sequences that did not have an *A. thaliana *homolog above the threshold were annotated with the best homolog match from a BlastX search versus the nr database at NCBI. Again an *E *value of < 10^-10 ^was used.

### UniGene set determination

Non-redundant sets of genomic singlets and contigs (UniGene sets) were created in a two-step procedure. First sequence information derived from rRNA, chloroplastic DNA or mitochondrial DNA was identified by comparison to homologous *Arabidopsis *sequences (accession nr. X52322, AP000423, and Y08501/Y08502 respectively) using BlastN. In this way, sequences containing rRNA or mitochondrial DNA were separated from the genomic sequences.

The second step was a BlastX search of the non-redundant (nr) protein database. The accession numbers and *E *values of the best matches were extracted from the result file. The criterion used for a sequence to be identified as non-redundant was based on a unique best match based on the accession numbers from the BlastX search. If two or more query sequences resulted in best matches with identical accession numbers they were sorted according to their *E *values. Only the sequence with the lowest *E *value was included in the UniGene set.

### Annotation and functional classification

The UniGene sets were annotated based on the results of BlastX searches of the nr database. The definition line of the Blast match was used as a description of the putative function of the UniGene gene. An *E *value threshold of 10^-10 ^was used and UniGene genes that did not meet this requirement were annotated as unknown.

Our functional classification of individual genes followed the functional categories as defined in the Munich Information Centre for Protein Sequences (MIPS) *Arabidopsis thaliana *functional catalogue (MATDB; downloaded from ). To create a semi-automated functional classification pipeline a two-step procedure was developed. First, a BlastX search was performed with the UniGene set versus the MATDB, requiring an *E *value of < 10^-10^. Locus name and *E *value of the best match for each gene were extracted from the result file. Secondly, the functional classification was identified by a search with the locus name in the *Arabidopsis *functional catalogue. Genes that did not meet the criteria for being functionally classified based on the semi-automated procedure were classified manually based on the annotation and the result from a conserved domain search versus the conserved domain database (CDD) downloaded from the NCBI web site.

### Identification of microsatellite sequences

A set of 3716 sequences resulting from the clustering and assembly steps, comprising a total of 5.3 Mb of sequence, was searched for microsatellites (simple sequence repeats; SSRs) in the form of mononucleotide repeats of > 15 bp, dinucleotide repeats of > 14 bp, trinucleotide repeats of > 15 bp, tetranucleotide repeats of > 16 bp, and pentanucleotide repeats of > 20 bp, as previously described by [29]. To better locate di- to pentanucleotide repeats, we also used the program Sputnik, developed at the Washington University [57]. This program allows minor imperfections on the SSRs by implementing a scoring system for insertions, mismatches and deletions. To locate mononucleotide repeats we used a simple PERL script developed by ourselves.

### RT-PCR

Reverse Transcriptase Polymerase Chain Reactions (RT-PCR) were performed on total RNA prepared from leaves of three weeks old oat plants (variety Gerald), incubated for 0 min, 15 min, 30 min, 45 min, 1 h, 2 h, 8 h and 24 h at +4°C, using the SuperScript™ III One-Step RT-PCR system (Invitrogene™). RNA samples were first DNase treated using the *Dnase I Amplification Garde *from Invitrogene™. To amplify the different *AsCBF *genes the following primers were used:

*AsCBF1 *forward primer 5'-CCACAGTCCACCGTATCAGCAAG-3'

*AsCBF1 *reverse primer 5'-CGTCTCCTTGAACTTGGTGCG-3'

*AsCBF3 *forward primer 5'-CGGGCAAAGTTGAGGCAGGC-3'

*AsCBF3 *reverse primer 5'-TAGGCTCTGGCTCGGCACCTTC-3'

*AsCBF4 *forward primer 5'-CCCAGCCTTCAGCAGCGTC-3'

*AsCBF4 *reverse primer 5'-TCTCCACAGTCTCCTCCGTGC-3'

For the *AsCBF1 *gene a product size of 174 bp was expected, for *AsCBF3 *104 bp and for *AsCBF4 *172 bp. The *AsActin *gene used as a control and was amplified using the forward primer 5'-GCGACAATGGAACTGGC-3 and the reverse primer 5'-GTGGTGAAGGAGTAACCTCTCTCG-3'. In this case the expected product size was 580 bp. The RT-PCR reactions were run according to the manufacturer's instructions and 100 ng total RNA were used in each reaction. A 30-min reverse transcription at 55°C followed by a PCR amplification step with 30, 35 or 40 cycles were used. To verify the outcome of the RT-PCR reactions, equal amounts (30%) of the corresponding RT-PCR reaction mixes were applied on 1% agarose gels containing ethidium bromide (0.5 ng per ml).

## Authors' contributions

MB contributed with EST data analysis, phylogenetic analysis, CBF expression experiments and with writing of the paper. AL did the data analysis on the most expressed genes. GC grew oat plants and performed the freezing experiment. SZ did cold induction of oat, RNA preparation, and quality and induction control of the RNA used for cDNA preparation. BO did EST data analysis, SSR identification and writing. OO contributed with planning, supervising and financing of the work and with writing of the paper.

## Figures and Tables

**Table 1:** Cold acclimation in oat. Frost damage in non-acclimated (20°C) and acclimated (12 or 24 h at +4°C) oat spring varieties Birgitta and Matilda and winter varieties Gerald and 83-48-CH. Plants were incubated at -15°C for 3 h, 6 h and 12 h and then recovered for 1 week in the greenhouse. Frost damage was recorded on a scale from 1 to 5, where 1 represents no damage and 5 represents dead plants.

	**-15°C incubation**		
**No acclimation**	**3 h**	**6 h**	**12 h**

Birgitta	2	5	5
Matilda	3	5	5
Gerald	1	3	5
83-48-CH	1	5	5
			
**12 h acclimation**	**3 h**	**6 h**	**12 h**

Birgitta	3	4	5
Matilda	3	4	5
Gerald	1	2	5
83-48-CH	1	2	5
			
**24 h acclimation**	**3 h**	**6 h**	**12 h**

Birgitta	3	3	5
Matilda	3	4	5
Gerald	1	2	5
83-48-CH	1	2	5

**Table 2:** The 20 most frequent, randomly picked, EST sequences in two different oat leaf libraries. Gene family, EST sequences identified as belonging to the indicated gene family; CI, total number of genes found in the indicated family in the cold induced leaf library; % of total, relative amount of genes in the indicated gene family, NI, total number of genes found in the indicated family in the non-induced leaf library.

**Gene family**	**CI**	**% of total**	**NI**	**% of total**
Chlorophyll a/b binding	952	11.3	39	1.8
Ribulose 1, 5-bisphosphate carboxylase/oxygenase small chain	657	7.8	210	9.6
Ribulose bisphosphate carboxylase/oxygenase activase	232	2.7	36	1.6
Carbonic anhydrase 2	149	1.8	29	1.3
Fructose-bisphosphate aldolase	144	1.7	2	0.1
Glyceraldehyde-3-phosphate dehydrogenase	107	1.3	6	0.3
Cold-induced COR410 (Wcor410)	82	1.0	0	0.0
Photosystem II oxygen-evolving complex (PsbP1)	58	0.7	11	0.5
LEA/RAB-related COR protein (Wrab17)	58	0.7	0	0.0
Photosystem II oxygen-evolving complex (PsbO2)	52	0.6	4	0.2
Ferredoxin	51	0.6	5	0.2
Photosystem II (PsbR)	50	0.6	4	0.2
Photosystem I reaction centre subunit XI	46	0.5	0	0.0
Glycolate oxidase	39	0.5	0	0.0
Alanine aminotransferase	39	0.5	3	0.1
Phosphoribulokinase	39	0.5	15	0.7
Aquaporin PIP	37	0.4	2	0.1
Glutamine synthetase	36	0.4	8	0.4
Hydroxyproline-rich glycoprotein	34	0.4	1	0.0
Photosystem I reaction centre subunit psaN	33	0.4	8	0.4

**Table 3:** Cold stress related oat genes. Distribution of genes in the AsCIUniGene set into different functional categories. Functional class, determined as described in Methods; AsCIUniGene set, the set of 2800 different oat genes; CSDB, set of cold related genes extracted from the public domain; % CSR, relative number of oat genes in each family that is similar to cold related genes.

**Functional class**	**AsCIUniGene set**	**CSDB**	**% CSR**
Cell cycle, DNA processing, cell fate and development	60	19	31.7
Cell rescue, defence and virulence	130	43	33.1
Cellular communication and signal transduction mechanisms	180	80	44.4
Cellular transport and transport mechanisms	138	28	20.3
Cellular organisation	31	5	16.1
Energy	215	5	2.3
Metabolism	431	87	20.2
Protein fate	144	13	9.0
Protein synthesis	93	1	1.1
Transcription	190	57	30.0
Unclassified protein	752	57	7.6
Unknown protein	427	2	0.5
Other	9	1	11.1

**Summary**	**2800**	**398**	**14.2**

**Table 4:** Percentage of cold stress related sequences in different UniGene sets. UniGene specifies the different UniGene sets, which were: AsCI, *Avena sativa *cold induced; AsNI, *Avena sativa *non-induced; TaCI, *Triticum aestivum *cold induced; HvCI *Hordeum vulgare *cold induced. Amount, refers to the number of sequences that were analysed. In CSDB, indicates how many of the total genes that also were present in the collection of cold stress related genes. Cold related (%), gives the percentage of genes in each set that were cold stress related.

**UniGene**	**Amount**	**In CSDB**	**Cold related (%)**
AsCI	2800	398	14.2
AsNI	1445	74	5.1
TaCI	2894	277	9.6
HvCI	3932	437	11.1

**Table 5:** Classification of oat transcription factors. Distribution of the 107 transcription factors, identified in the AsCIUniGene set, in different families classified according to Reichmann et al, 2000.

**Family**	**No of genes**
AP2/EREBP	11
bHLH	2
bZIP	9
CONSTANS B-box	5
DOF	3
Homeodomain	12
IAA	6
Leuzin zipper	3
MADS box	3
MYB	9
SCARECROW	3
WRKY	4
Zink finger	23
Other transcription factors	14
**Total**	**107**

**Table 6:** List of sequences containing the ERF/AP2 DNA-binding domain. The list of ERF/AP2 sequences used in the phylogenetic analysis (figure 4), some of the sequences were also used in the ClustalW alignment (figure 5). The first two letters in the protein name represent the initial letters of the Latin binomial, followed by the gene abbreviation. Each sequence is assigned to one of three different subgroups of the ERF/AP2 superfamily. *A. sativa *sequences are grouped according to our phylogenetic analysis (Figure 4). All GI accession numbers correspond to protein sequences in gene bank at NCBI [53], and the EMB accession numbers correspond to EST sequences available in the EMBL-nucleotide sequence database [58].

Species	Gene name	Group	Accessions	Reading farme^1^
Arabidopsis thaliana	AtABI4	DREB	gi|15225661	
	AtAPETALA2	AP2/RAV	gi|15234566	
	AtANT	AP2/RAV	gi|15235690	
	AtCBF1	DREB	gi|18416562	
	AtCBF2	DREB	gi|18416557	
	AtCBF3	DREB	gi|18416559	
	AtDREB2A	DREB	gi|15239107	
	AtDREB2B	DREB	gi|15228427	
	AtERF1	ERF	gi|3434967	
	AtERF2	ERF	gi|3434969	
	AtERF3	ERF	gi|3434971	
	AtERF5	ERF	gi|3434975	
	AtERF6	ERF	gi|3298498	
	AtLEAFY_PETIOLE	ERF	gi|15240749	
	AtRAP2.1	DREB	gi|18401592	
	AtRAP2.2	ERF	gi|18400321	
	AtRAP2.3	ERF	gi|15228312	
	AtRAP2.4	DREB	gi|2281633	
	AtRAP2.5	ERF	gi|2281635	
	AtRAP2.6	ERF	gi|15218275	
	AtRAP2.7	AP2/RAV	gi|18401775	
	AtRAP2.10	DREB	gi|15234561	
	AtRAP2.11	AP2/RAV	gi|15241182	
	AtRAP2.12	ERF	gi|15220971	
	AtRAV1	AP2/RAV	gi|25091118	
	AtRAV2	AP2/RAV	gi|11357264	
	AtTINY	DREB	gi|15239501	
Avena sativa	AsAP2.1	ERF	emb|AM071401	+ 1
	AsAP2.2	ERF	emb|AM071402	+ 2
	AsAP2.3	ERF	emb|AM071403	- 3
	AsAP2.4	AP2/RAV	emb|AM071404	+ 1
	AsAP2.5	AP2/RAV	emb|AM071405	+ 2
	AsCBF1	DREB	gi|72059436	
	AsCBF2	DREB	gi|72059440	
	AsCBF3	DREB	emb|AM071408	+ 2
	AsCBF4	DREB	gi|72059443	
	AsERF1	AP2/RAV	emb|AM071410	+ 2
	AsERF2	AP2/RAV	emb|AM071411	+ 2
Brassica napus	BnCBF1	DREB	gi|17352283	
	BnCBF2	DREB	gi|17352285	
Catharanthus roseus	CrORAC1	DREB	gi|8346773	
	CrORAC2	ERF	gi|8346775	
	CrORAC3	ERF	gi|8980315	
Hordeum vulgare	HvCBF1	DREB	gi|12658319	
	HvCBF2	DREB	gi|20152903	
	HvCBF3	DREB	gi|12658321	
Nicotiana tabacum	NtACRE111A	DREB	gi|12003382	
	NtACRE111B	DREB	gi|12003384	
	NtTSI1	ERF	gi|3065895	
Lycopersicon esculentum	LeCBF1	DREB	gi|18535580	
Oryza sativa	OsDREB1A	DREB	gi|22594969	
	OsDREB1B	DREB	gi|22594973	
	OsDREB2	DREB	gi|22594971	
Secale cereale	ScCBF	DREB	gi|17148651	
Triticum aestivum	TaDREB	DREB	gi|17226801	

1) Nucleotide sequences that have been used in the phylogenetic analysis or in the ClustalW alignment have been translated in to amino acid sequences using the indicated reading frames.
